# Fluorinated Polyurethanes, Synthesis and Properties

**DOI:** 10.3390/molecules21070904

**Published:** 2016-07-12

**Authors:** Olga Smirnova, Alexey Glazkov, Alexander Yarosh, Alexey Sakharov

**Affiliations:** N.D. Zelinsky Institute of Organic Chemistry of Russian Academy of Sciences, 47 Leninsky Avenue, Moscow 119991, Russian; smirnova@ioc.ac.ru (O.S.); as@ioc.ac.ru (A.G.); yar@ioc.ac.ru (A.Y.)

**Keywords:** aromatic and cycloaliphatic diisocyanates, fluorinated alcohols, fluorinated polyurethanes, glass transition temperature, decomposition temperature

## Abstract

Fluorinated polyurethanes with a glass transition temperature as low as −139 °C and a decomposition onset temperature of 247–330 °C were prepared by a reaction of fluorinated alcohols with aromatic and cycloaliphatic diisocyanates in solution or melt.

## 1. Introduction

Reactions of aromatic and aliphatic diisocyanates are extensively employed in the synthesis of valuable polymeric materials, viz. polyurethanes (PUs). The preparation of polymers is often assisted by catalysts such as tertiary amines, organotin compounds, etc. [1]. Depending on the starting compounds, plastics or rubberlike products are afforded.

The fluorine incorporation into the polymer backbone or its side chains is the basis for well known methods that lead to great changes in the surface properties [2]. PU is one material that would benefit from the characteristic properties intrinsic to fluorinated polymers. The effect of fluorine-containing PUs on their properties has been described [3,4,5,6,7]. Fluorocarbon chains were incorporated into PUs via fluorine-containing isocyanates [8], chain extenders [3,4,5,6,7] or soft segments [9,10,11]. The surface and bulk structure of the synthesized PUs were studied. Ge at al. [12] synthesized FPUs starting from fluorinated polyether glycol prepared by radical grafting of hexafluoropropylene onto polytetramethylene glycol (PTMG-*g*-HFP) as a soft segment, 1,6-hexamethylene diisocyanate or toluene diiscyanate as a hard segment and 1,4-butanodiol as a chain extender. They studied their mechanical properties and thermal stability in comparison with those of corresponding hydrogenated PUs along with chemical resistance of FPU films through spot tests with different solvents.

It was also shown that introduction of fluorinated fragments to a macromolecule in addition to high hydro- and oleophobic properties considerably contribute towards achievement by the prepared polymers good low temperature parameters in a few cases [13]. 1,4-Butanediol, 2,2,3,3,4,4,5,5-octafluoro-1,6-hexandiol, etc., are often utilized as macromolecule chain extenders [14,15,16,17,18]. Thus, in a number of cases, the glass transition temperature of polymers can be dramatically reduced by the insertion of fluorinated fragments to the macromolecule [19,20].

**Scheme 1:**
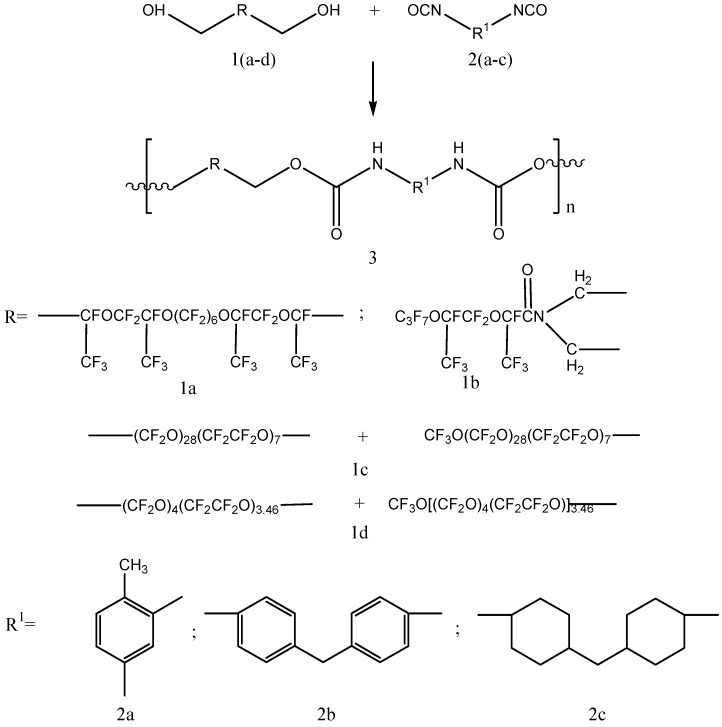
FPUs synthesis pathway.

In this research we endeavored to synthesize new poly- and oligourethanes containing fluoropolyether components of different structure both in the backbone and in the side polymer chain. Aromatic and cycloaliphatic diisocyanates were used as hard segments of FPUs. A synthetic route to such FPUs is given in Scheme 1.

## 2. Results and Discussion

Polyaddition of diisocyanates to fluorinated alcohols was performed both in solution (dimethylformamide (DMFA) or perfluorobenzene) and in mass (melt) at temperatures varying from 25 °C to 190 °C. Ethylene glycol was utilized as a chain extender in a number of tests. The synthetic conditions are specified in Table 1.

Analyzing the course of the synthesis of FPUs from aromatic and cycloaliphatic diisocyanates, we established that the polyaddition reaction proceeded quite smoothly without a catalyst both in solvents and in the melt. From technological and environmental points of view, the bulk process applied in this research could have the certain advantage against the process in solution. Meanwhile, a number of researchers explained a need for a catalyst by low basicity of fluorinated diols. Apparently, reactivity of fluorinated diols in such reactions does not merely rely on their basicity.

**Figure 1:**
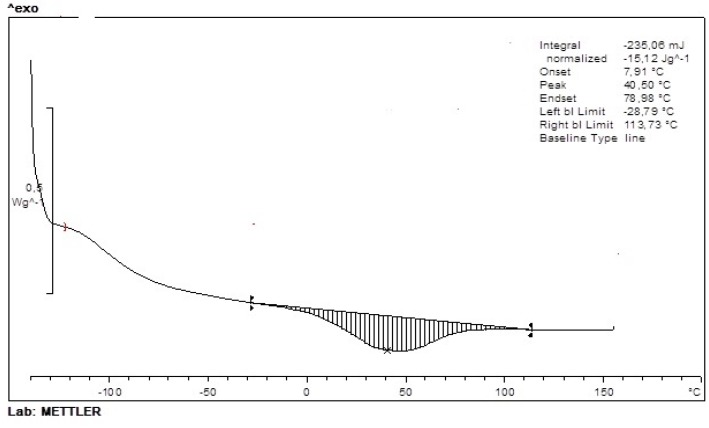
Differential scanning calorimetry (DSC) data for fluoropolyurethane **5**.

Figure 1 shows DSC data for fluoropolyurethane **5**, which evidence that the prepared low molecular weight polymer has a partially crystalline structure. DSC curve shows temperature interval from −30 °C to 115 °C corresponding to melting procedure with maximum at 40 °C. Phase regularity of polymer chains is likely to be related to interactions between neighboring macromolecules containing urethane groups.

**Figure 2:**
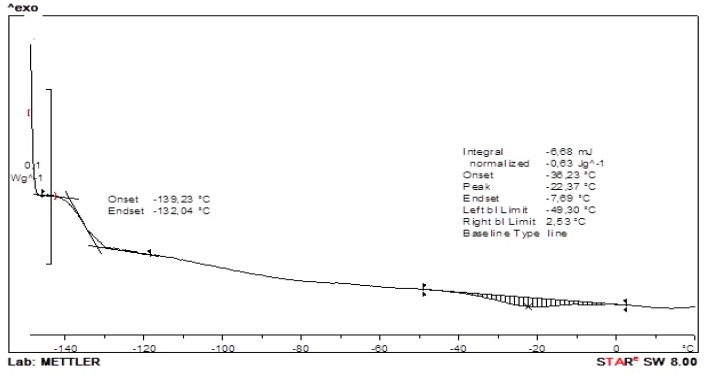
DSC data for fluoropolyurethane **7**.

Figure 2 illustrates DSC data for rubberlike high molecular weight FPU **7** obtained from diol **1d** and diisocyanate **2c** in melt. The glass transition temperature of FPU **7** is −139 °C. An insignificant share of the crystalline phase with the melting point −22 °C was observed herein. It can be concluded that FPU **7** is characterized by a relatively weak intermolecular interaction between C=O and N-H groups of macromolecules. This FPU dissolved only in C_6_F_6_, did not dissolve in DMFA and, to some extent, dissolved in acetone.

**Figure 3:**
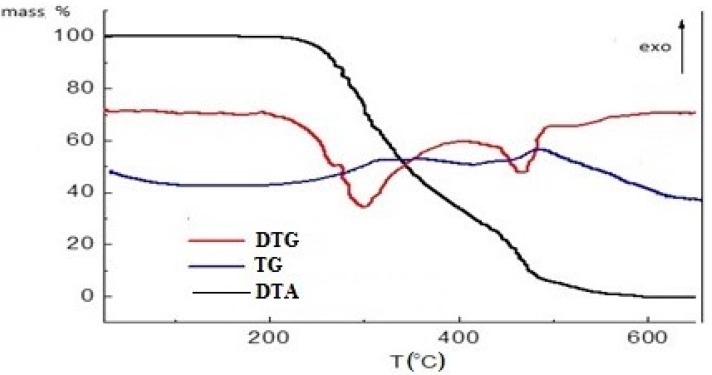
Thermogravimetric analysis (TGA) data for fluoropolyurethane **7**.

Figure 3 provides thermogravimetric analysis (TGA) data obtained in argon for this polymer. TGA data indicate that the beginning temperature of polymer destruction is close to 300 °C and maximum rate of destruction is achieved at 460 °C. The TGA results for all other polymers were actually similar and didn′t depend on measurement conditions—either in argon or air flow.

Summarizing DSC and TGA data for the selected FPUs synthesized in this research (Table 1), it might be concluded that the temperature interval of FPUs performance (−139 °C to 247–330 °C) is much broader than that of analogous hydrogenated PUs which is normally within −60 to 160 °C depending on the structure of glycols and diisocyanates used as starting materials. Thus, the decomposition onset temperature of PUs prepared from alkylendiisocianates and alkylenglycols is around 250 °C, of PUs from aromatic diisocyanate-alkylene glycol 200 °C, and from alkylenediisocyanate-aromatic glycols 180 °C [21,22,23]. Glass transition temperature (T_g_) of these PUs were found to be approximately −60 °C.

The measurement of contact angles on the water-wetted surface of the prepared FPUs was performed as follows. The attained FPU melts were applied to glass plates that were then heated to 90 °C in a desiccator until evaporation of the solvents. Transparent or semitransparent films were prepared. Contact angles of their surface wetting with water were determined. The contact angles achieved 100°−117° (Table 1), which was indicative of rather high hydrophobicity of the coatings.

Syntheses of all other tabulated FPUs were performed in the conditions summarized in Table 1. At rather low temperatures FPUs were synthesized in solution. At high temperatures, the polyaddition process was conducted in the melt. Initial mixture composition includes aromatic diisocyanates (rows 1–6) and cycloaliphatic ones (rows 7–12). Depending on the starting materials and reaction conditions used, the oligomers and polymers obtained exhibit different properties. Some of them (like polymer **5**, Figure 1) possess crystal segments of polymer chain, others are rubberlike amorphous materials (7–12 samples). Regardless the difference in structure and properties, all of them were shown to possess pronounced hydrophobic properties (surface wetting contact angles found were higher than 100°).

## 3. Materials and Methods

Diisocyanates **2a**–**c** were commercial products (manufacturer Bayer Material Science) pre-dried in vacuo at 100−150 °C: **2a** M = 174.2, bp = 251 °C; **2b** M = 250.25, bp = 152−154 °C (3 Torr); **2c** M = 262.35, bp = 358 °C.

Fluorinated alcohols **1a**–**d** incorporating fluoropolyether units were used as hydroxymethylene derivatives: **1a** M = 902, bp = 146−150 °C (0.01 Torr), **1b** M = 595, bp = 210 °C (0.01 Torr), **1c** M = 2442, and **1d** M = 1418. Bp of the products was above 200 °C (0.01 Torr). They were prepared according to the procedure reported in [24] and represent fluoropolyether glycols or fluoropolyether amides containing hydroxymethylated terminal groups (**1a**–**b**) or a mixture of fluoropolyether glycols with alcohols of similar structure (**1c**–**d**) with ratio 7:1. Molecular weights and compositions of the prepared fluoropolyether diols and alcohols were found from ^19^F-NMR and ^1^H-NMR data. ^1^H-NMR and ^19^F-NMR were using a Bruker AM 300 SF at 300 MHz (^1^H), 282,40 MHz (^19^F). ^1^H-NMR chemical shifts were referenced to the residual proton signal from CDCl_3_ served as an internal standard. ^19^F-NMR spectra (solutions in C_6_F_6_, −163 ppm) served as an inside standard. Multiplicities are reported as singlet (s), doublet (d), triplet (t) and some combinations of these, multiplet (m).

The molecular weight characteristics of the polymers were determined using the gel permeation chromatography (GPC) method in DMFA at 35 °C on a Knauer liquid chromatograph equipped with a refractometer RI-2300 and three columns (Waters Styrogel HT-2, HT-4, and HT-6E). The elution rate 1 mL/min. The calibration was done using the polystyrene standard (PL Polymer Laboratories) in the range from 1250 to 1030000 Da.

The glass transition temperature was determined by the differential scanning calorimetry (DSC) on a DSC-822e instrument (Mettler Toledo, Switzerland) using ~10 mg samples at the heating rate 10 °C/min. IR spectra were registered on a Specord IR-75 spectrometer.

TGA data were determined on the instrument Derivatograph-C (MOM, Hungary) in air and argon flow using ~15 mg samples at the heating rate 10 °C/min.

Inherent viscosity was determined using an Ubbelohde viscometer in DMFA or C_6_F_6_.

### 3.1. Synthesis of Fluorinated Alcohol ***1b***

Pre-distilled diethanolamine (3.32 g, 0.032 mol), bp = 122–125 °C (3 Torr) was placed in a round bottomed flask fitted with a stirrer, thermometer and drop funnel and then 16.2 g (0.032 mol) methyl ester of the hexafluoropropylene oxide trimer was added dropwise from the funnel. The reaction mixture was stirred for 2 h at 65 °C until homogeneity. In the IR spectrum of the products, a signal in the region of 1780 cm^−1^ disappeared (C=O in ester) and a signal in the region of 1740 cm^−1^ emerged (C=O in amide). ^1^H-NMR, (CDCl_3_) δ: 4.38 (2H, s, OH); 2.26 (4H, t, O=C-CH_2_CH_2_); 3.18 (t, 4H, CH_2_OH); ^19^F-NMR, (C_6_F_6_) δ: −80.0 to −88.0 (m, 13F CF_2_O + CF_3_); −126.0 to −127.4 (s, 1F OCF(CF_3_)C); −132.0 (m, 2F CF_3_CF_2_CF_2_); −147.0 (m, 1F CF_2_CF(CF_3_)C=O).

### 3.2. Synthesis of Fluoropolyurethanes

#### 3.2.1. Synthesis of Fluoropolyurethane **5**

Diol **1b** (1.88 g, 0.032 mol) in 10 mL DMFA was placed in a four-neck flask fitted with a stirrer, drop funnel, thermometer, and dry nitrogen feeder and then 0.76 g (0.03 mol) of diisocyanate **2b** was added. The process was conducted with intensive stirring in the dry nitrogen flow during 10 h. After DMFA evaporation, in the waterjet pump vacuum was prepared a solid, brittle and transparent mass M_w_ = 9667; M_n_ = 6062, D = 1.59–1.60 (Figure 1), mp = 95–98 °C (Figure 2), T_5% decomp._ = 247 °C.

The IR spectrum, (KBr), cm^−1^, ν: 3338 (NH), 3032 (ArH), 2923 (CH), 1719 (C=O), 1601 (C-C arom.), 1541 (NH), 1226, 1046 (CF). ^1^H-NMR, (CDCl_3_) δ: 6.04 (8H-arom. s.); 3.83−3.74 (4H, t, CH_2_); 2H (Ar-CH_2_ Ar, s,). ^19^F-NMR, (C_6_F_6_,) δ,: −80.0 to −88.0 (13F, m, CF_2_O+CF_3_); −126.0 to −127.4 (1F, s, OCF(CF_3_)C=O); −132.0 (2F, m, CF_3_CF_2_ CF_2_); −147.0 (1F, s, OCF_2_CF(CF_3_)O).

#### 3.2.2. Synthesis of Fluoropolyurethane **7**

Diol **1c** (2.46 g, 0.001 mol) pre-heated at 110 °C (18 Torr) during 2 h was placed in a four-neck flask fitted with a stirrer, drop funnel, thermometer, and dry nitrogen feeder and then 0.27 g (0.001 mol) of diisocyanate **2c** was added. The process was conducted with intensive stirring in the dry nitrogen flow, gradually increasing the temperature from 25 °C to 190 °C, during 50 h. A homogeneous, thick, colorless and transparent mass was prepared. The reaction completion was monitored against the absence of the free NCO group absorption band in the region of 2270 cm^−1^, T_g_ = −139 °C (Figure 2), T_5% decomp._ = 300 °C. Maximum decomposition rate was ≈ 450 °C (Figure 3).

The IR spectrum of fluoropolyurethane **7**, (KBr), cm^−1^, ν: 3337 (NH); 3032 (CH arom.), 2923 (CH); 1719 (C=O, uretane); 1534 (NH); 1226, 1046 (C-F). ^1^H-NMR (CDCl_3_,) δ: 1.32 (8H, m, CH_2_-CH_2_); 1.70 (8H, t, CH_2_-N); 4.47 (4H d, R_F_CH_2_O); 5.55 (2H s, NH). ^19^F NMR (C_6_F_6_,) δ: −52.16 (3F, s, CF_3_O); −52.76 to −54.71 (2F, s, (CF_2_O)_n_); −88.0 to −89.7 (4F, d, (CF_2_CF_2_O)_n_).

## 4. Conclusions

Novel FPUs possessing promising performance properties have been synthesized starting from fluorinated diols as soft segments and both aromatic and cycloaliphatic diisocyanates as hard segments. Values of their glass transition temperature and the beginning temperature of thermal decomposition achieve −139 °C and 247–330 °C, correspondingly, showing their performance in quite wide temperature intervals in comparison to non-fluorinated analogs. According to literature data, the temperature interval of hydrogenated PUs performance is predominately in the range from −60 °C to 160 °C.

Contact angles of surface wetting with water for FPUs obtained reach 100–117°, providing evidence of quite favorable hydrophobic properties of fluorinated polyurethanes. Potentially, novel fluorinated polyurethanes can be used for production of materials exploited under extreme conditions and in compositions of complex organic and hybrid molecular systems [25,26].

## Figures and Tables

**Table 1:** Fluoropolyurethane synthesis conditions and some parameters of the prepared polymers.

No.	Initial Mixture Composition	Reaction Conditions	Polymer′s Appearance	Contact Angle by Water (°)	Parameters of the Polymers
1	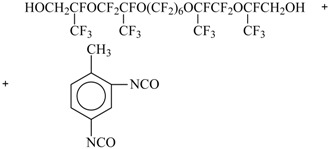	60 °C–150 °C 17 h in nitrogen flow DMFA	Solid elastic light-brown	101	(η) = 0.006 dL/g in DMFA
2	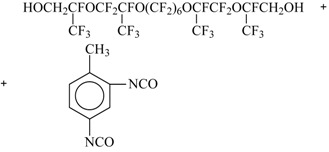	190 °C 17 h in nitrogen flow, in melt	Solid elastic light-brown	101	Mp = 20 °C; (ƞ) = 0.01 dL/g in DMFA T_5% decomp._ = 261 °C
3	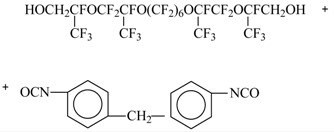	190 °C 15 h in nitrogen flow, in melt	Solid yellowish brown	110	T_5% decomp._ = 323 °C
4	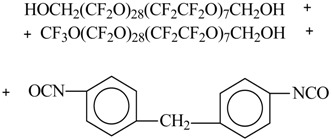	190 °C 9 h in nitrogen flow, in melt	Colorless transparent	113	T_5% decomp_. = 247 °C T_g_ = −143.6 °C (η) = 0.13 dL/g in C_6_F_6_
5	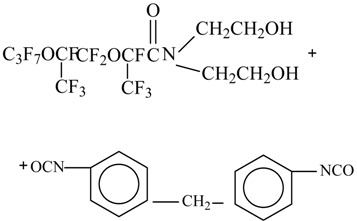	Room temp., at stirring, in nitrogen flow 1.05:1 DMFA	Brittle yellowish mass	111	Mp = 95−98 °C T_5% decomp._ = 247 °C М_W_ = 9667 М_n_ = 6062 D = 1.59–1.60
6	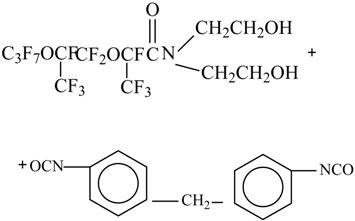	Room temp., at stirring, in nitrogen flow, 1:1.1 DMFA	Brittle yellowish-brown mass	114	Mp = 94–96 °C
7	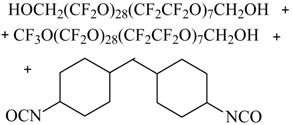	until 190 °C in nitrogen flow 50 h, 1:1, in melt	Homogenous thick colorless transparent mass	102	(η) = 0.14 dL/g T_g_ = −139 °C Mp = − 22 °C (contains cryst.polym. segments) T_5% decomp._ = 300 °C
8	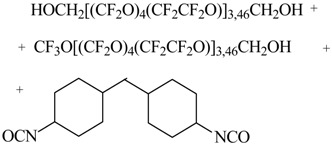	until 190 °C in nitrogen flow 16 h, 1:1, in melt	Homogenous elastic opalescent mass	104	(η) = 0.103 dL/g T_g_ = −132 °C Mp = −12 °C (contains cryst. polym. segments) T_5% decomp._ = 290 °C
9	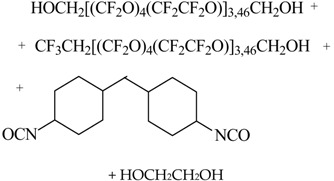	60 °C in nitrogen flow, at stirring 10 h 1:3:2 C_6_F_6_	Homogenous white viscous elastic mass	103	T_g_ = −132 °C
10	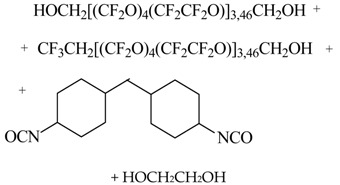	60 °C in nitrogen flow, at stirring 7 h 1:2:1 C_6_F_6_	Homogenous white solid elastic mass	106	T_g_ = −136 °C
11	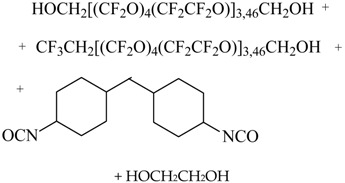	60 °C in nitrogen flow, at stirring 12 h 1:2:5 C_6_F_6_	Homogenous white elastic mass	117	(η) = 0.14 dL/g T_g_ = −133 °C T_5% decomp._ = 244 °C
12	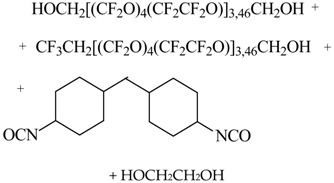	until 190 °C in nitrogen flow 7 h 1:2:5 in melt	Homogenous beige solid elastic mass	106	T_g_ = −137 °C T_5% decomp._ = 237 °C
